# Comparison of Traditional Chinese Exercise and Equipment‐Based Exercise on Glycaemic Control and Vitamin D Levels in Middle‐Aged and Elderly Patients With Prediabetes: A Randomised Controlled Trial

**DOI:** 10.1002/edm2.70326

**Published:** 2026-09-08

**Authors:** Xu Lingzhi, Zhou Chunhua, Liu Wu, Ding Jiejin, Yuan Yimin

**Affiliations:** ^1^ Health Management Center Wuxi Hospital of Traditional Chinese Medicine Wuxi Jiangsu Province China

**Keywords:** elderly, exercise therapy, glucose metabolism, middle‐aged, prediabetes, randomised controlled trial, traditional Chinese exercise, vitamin D

## Abstract

**Background:**

Prediabetes is a critical window for preventing progression to type 2 diabetes mellitus (T2DM). Both vitamin D deficiency and physical inactivity are associated with impaired glucose metabolism. This study compared the effects of Traditional Chinese Exercise (TCE) and Kuanle Equipment Exercise (KEE) on glycaemic measures, lipid profiles, body composition and serum 25‐hydroxyvitamin D_3_ [25(OH)D_3_] in middle‐aged and elderly adults with prediabetes.

**Methods:**

We conducted a 12‐week, single‐centre, randomised controlled trial involving 62 participants with prediabetes (mean age 55.2 ± 8.4 years; 88.7% female). Participants were randomly assigned to control (usual care, *n* = 21), TCE (*n* = 20), or KEE (*n* = 21). Primary outcomes included fasting plasma glucose (FPG), 2‐h OGTT glucose, glycated haemoglobin (HbA1c) and serum 25(OH)D_3_. Secondary outcomes comprised triglycerides, total cholesterol, LDL‐C, HDL‐C and body composition.

**Results:**

At baseline, mean serum 25(OH)D_3_ was 21.53 ± 3.90 nmol/L. At 12 weeks, both TCE and KEE improved FPG, 2‐h OGTT glucose, HbA1c and 25(OH)D_3_ versus control (all *p* ≤ 0.001). Relative changes in FPG were −9.4% (TCE) and −15.2% (KEE) versus −1.5% (control); corresponding changes were −14.6% and −21.2% for 2‐h OGTT glucose, −6.7% and −12.9% for HbA1c, and +60.6% and +81.9% for 25(OH)D_3_. Direct TCE–KEE comparisons were not significant (all *p* > 0.05). In an exploratory analysis, change in 25(OH)D_3_ was inversely associated with change in 2‐h OGTT glucose (partial *r* = −0.358, *p* = 0.006), but not with FPG, HbA1c, or triglycerides.

**Conclusions:**

Both TCE and KEE improved glycaemic outcomes and increased serum 25(OH)D_3_ compared with usual care in middle‐aged and elderly adults with prediabetes. Although KEE produced numerically larger estimates, direct between‐group comparisons were not significant, and the superiority of KEE over TCE was not established. These vitamin D findings require confirmation in trials that quantify sunlight exposure and dietary vitamin D intake.

## Graphical abstract

In middle‐aged and elderly patients with prediabetes, both Traditional Chinese Exercise (Baduanjin) and equipment‐based exercise improved glycaemic control and significantly raised serum 25(OH)D_3_ (+60.6% to +81.9%) over 12 weeks. KEE showed larger numerical changes, but direct comparison did not establish superiority over TCE.

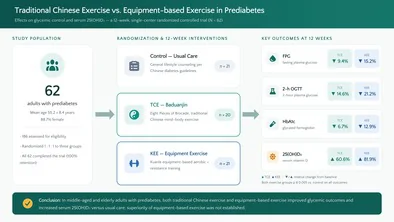

## 1 Introduction

Type 2 diabetes mellitus (T2DM) has emerged as a global public health crisis, affecting approximately 537 million adults worldwide with projections suggesting 783 million cases by 2045 [1]. Prediabetes, characterised by elevated blood glucose levels that do not meet diagnostic criteria for diabetes, represents a critical transitional state with an annual progression rate to T2DM of 5%–10% [2]. However, this trajectory is not inevitable—lifestyle interventions have demonstrated the potential to reduce diabetes incidence by up to 58% [3].

The pathophysiology of prediabetes involves insulin resistance, β‐cell dysfunction and chronic low‐grade inflammation [4]. Among modifiable risk factors, vitamin D deficiency has garnered substantial attention due to its pleiotropic effects on glucose homeostasis. The active metabolite, 1,25‐dihydroxyvitamin D, regulates insulin secretion through direct actions on pancreatic β‐cells and improves insulin sensitivity via effects on skeletal muscle and adipose tissue [5]. Epidemiological studies consistently demonstrate inverse associations between serum 25‐hydroxyvitamin D [25(OH)D] concentrations and T2DM risk [6].

Physical exercise remains the cornerstone of prediabetes management. Regular aerobic exercise enhances insulin sensitivity through multiple mechanisms: increased GLUT4 translocation, improved mitochondrial function, reduced visceral adiposity and modulation of inflammatory cytokines [7]. Resistance training offers additional benefits by increasing muscle mass—the primary site of glucose disposal—and improving basal metabolic rate [8].

Traditional Chinese exercises, including Tai Chi, Qigong and Baduanjin, have demonstrated particular promise for middle‐aged and elderly populations due to their low‐impact nature and integration of physical movement with mindful breathing. These modalities improve balance, reduce falls and enhance psychological well‐being while conferring metabolic benefits [9]. Conversely, modern equipment‐based exercise programs offer standardised, quantifiable training stimuli that may optimise physiological adaptations through progressive overload principles [10].

The prevalence of prediabetes increases markedly after age 45, affecting both middle‐aged adults (45–59 years) and elderly individuals (≥ 60 years). Both groups face common metabolic challenges including declining physical activity, age‐related sarcopenia and high rates of vitamin D deficiency, yet their exercise preferences and physical capacities may differ substantially [11]. Vitamin D deficiency is highly prevalent among Chinese populations in these age groups, with reported rates exceeding 70% in urban settings [11]. Contributing factors include reduced cutaneous synthesis with aging, limited sun exposure, dietary patterns low in vitamin D‐rich foods and increased adiposity that sequesters vitamin D in adipose tissue [12]. Importantly, according to the Institute of Medicine, 25(OH)D concentrations below 30 nmol/L indicate risk of deficiency, levels of 30–50 nmol/L may be inadequate for some individuals and ≥ 50 nmol/L are considered sufficient for most people [13].

Emerging evidence suggests that exercise may influence vitamin D metabolism through enhanced hepatic hydroxylation, altered vitamin D binding protein kinetics and reduced sequestration in adipose tissue due to fat loss [14]. However, the interaction between exercise modality and vitamin D levels in middle‐aged and elderly prediabetic individuals remains inadequately characterised.

Given the high prevalence of prediabetes and vitamin D deficiency among middle‐aged and elderly Chinese populations, structured exercise may offer a practical approach to improving metabolic health. This randomised controlled trial was designed to: (1) evaluate and compare the effects of TCE and KEE on glycaemic control; (2) assess changes in serum 25(OH)D_3_ concentrations after each exercise intervention; and (3) explore whether within‐person changes in 25(OH)D_3_ were associated with changes in metabolic outcomes. The study evaluated the two exercise programs separately and did not test a combined TCE‐plus‐KEE intervention.

## 2 Materials and Methods

### 2.1 Study Design and Setting

This study was a 12‐week, single‐centre, randomised controlled trial conducted at the Health Management Center of Wuxi Hospital of Traditional Chinese Medicine, Jiangsu Province, China, from July 2024 to December 2024. The study protocol was approved by the Institutional Review Board of Wuxi Hospital of Traditional Chinese Medicine (IRB approval number: 20240326), and all participants provided written informed consent before enrollment. The trial was filed with the National Medical Research Registration and Filing Information System (MR‐32‐24‐047398) before enrollment; retrospective registration with ChiCTR is pending review (Supporting Information S1).

### 2.2 Participants

Eligible participants were community‐dwelling adults meeting the following inclusion criteria: (1) age ≥ 45 years; (2) prediabetes diagnosis according to American Diabetes Association criteria—fasting plasma glucose (FPG) 5.6–6.9 mmol/L, impaired glucose tolerance (2‐h OGTT glucose 7.8 to < 11.1 mmol/L), or glycated haemoglobin (HbA1c) 5.7%–6.4%; (3) ability to participate in structured exercise programs; and (4) provision of written informed consent.

Exclusion criteria included: (1) diagnosed diabetes mellitus requiring medication; (2) significant cardiovascular, renal, or hepatic disease; (3) musculoskeletal conditions precluding exercise participation; (4) use of vitamin D supplements exceeding 800 IU/day within the preceding 6 months or current use of glucose‐lowering medication; (5) participation in a structured exercise program within the preceding 6 months; and (6) cognitive impairment affecting adherence. The 6‐month vitamin D exclusion window was selected conservatively and exceeded approximately five half‐lives of circulating 25(OH)D, thereby reducing—although not eliminating—the likelihood of residual supplementation effects.

The duration of prediabetes was not systematically recorded because a substantial proportion of participants were newly identified through community screening or lacked a clearly documented date of initial diagnosis.

### 2.3 Randomisation and Blinding

Participants were randomly assigned to one of three groups using computer‐generated randomisation sequences with block sizes of 6 and allocation ratio 1:1:1. Allocation concealment was achieved using sequentially numbered, opaque, sealed envelopes. Due to the nature of exercise interventions, participants and exercise instructors could not be blinded to group assignment. However, outcome assessors and laboratory personnel were blinded to group allocation.

### 2.4 Interventions

#### 2.4.1 Control Group

Participants in the control group received usual care consisting of general lifestyle counselling based on Chinese Diabetes Society guidance [15]. The physical activity guidance included at least 150 min/week of moderate‐intensity aerobic activity (e.g., brisk walking) and resistance exercise involving major muscle groups 2–3 times/week. This advice was educational rather than a supervised or structured exercise intervention. The dietary component was limited to general healthy‐eating advice; no individualised calorie restriction, vitamin D dietary prescription, or standardised diet was implemented. Participants in all three groups were instructed to maintain their habitual dietary patterns throughout the 12‐week study.

#### 2.4.2 Traditional Chinese Exercise (TCE) Group

The TCE intervention comprised Baduanjin (Eight Pieces of Brocade), a classical Qigong exercise system consisting of eight standardised movements combining gentle stretching, controlled breathing and meditative focus. Sessions were conducted three times weekly (Monday, Wednesday, Friday) for 45 min under certified Baduanjin instructor supervision (National Health Qigong Management Center, China). Each session included a 5‐min warm‐up, 30‐min Baduanjin practice (3 cycles of 8 movements) and a 10‐min cool‐down.

#### 2.4.3 Kuanle Equipment Exercise (KEE) Group

The KEE intervention utilised specialised resistance and aerobic training equipment (Kuanle Health Technology Co. Ltd., China) designed for elderly users. Sessions followed a structured protocol: 5‐min warm‐up, 30 min of circuit training incorporating leg press, chest press, rowing and stationary cycling at 60%–70% of heart rate reserve, and 5‐min cool‐down. Training frequency was three sessions weekly under ACSM‐certified exercise specialist supervision.

#### 2.4.4 Standardisation and Compliance

All exercise sessions were conducted at the Health Management Center. Attendance was recorded, and adherence was defined as completion of at least 80% of the 36 scheduled sessions. Participants were instructed to avoid additional structured exercise and to maintain their habitual dietary patterns, outdoor activity and sunlight exposure. No advice was given to increase sunlight exposure, alter dietary vitamin D intake, or initiate vitamin D supplementation. Monthly telephone follow‐up was conducted for all participants to monitor adverse events and consistency of habitual behaviours; exercise‐group participants also received weekly reminders regarding scheduled sessions.

### 2.5 Outcome Measures

#### 2.5.1 Primary Outcomes

Blood samples were collected following overnight fasting (≥ 8 h) at baseline and Week 12. Serum 25(OH)D_3_ concentrations were measured using chemiluminescent immunoassay (Roche Diagnostics, Switzerland). The intra‐assay and inter‐assay coefficients of variation (CV) for 25(OH)D_3_ measurement were 4.2% and 6.8%, respectively. Glucose metabolism parameters included FPG and 2‐h OGTT glucose (measured via oral glucose tolerance test with 75 g glucose load) using hexokinase method and HbA1c via high‐performance liquid chromatography (TOSOH G8 Analyser, Japan). The HbA1c assay is NGSP‐certified (Level I Laboratory certification). Quality control was performed daily using commercial control materials at two concentration levels, with all control values within manufacturer‐specified ranges throughout the study period.

#### 2.5.2 Secondary Outcomes

Lipid profiles comprised triglycerides (TG), total cholesterol (TC), high‐density lipoprotein cholesterol (HDL‐C) and low‐density lipoprotein cholesterol (LDL‐C) measured via enzymatic methods. Body composition was assessed using bioelectrical impedance analysis (InBody 770, South Korea), including body mass index (BMI), body fat percentage and skeletal muscle mass.

### 2.6 Statistical Analysis

Sample size was planned around a 0.4%‐point between‐group difference in HbA1c, informed by meta‐analytic evidence on structured exercise [16]. Assuming a two‐sided α of 0.05, 80% power and 15% attrition, the target sample was 30 participants per group (*n* = 90). Recruitment constraints limited enrollment to 62 participants (20–21 per group). With these final group sizes, a two‐sided pairwise comparison had 80% power to detect a standardised difference of approximately 0.90; assuming an HbA1c SD of 0.4%, the prespecified 0.4% difference (standardised effect *d* = 1.0) corresponded to approximately 88% power. The study remained underpowered for smaller effects and subgroup analyses.

Statistical analyses were performed using SPSS version 26.0 (IBM Corp., USA). All randomised participants were analysed according to assigned group. Continuous variables are presented as mean ± SD and categorical variables as *n* (%). Distributional assumptions were evaluated using Shapiro–Wilk tests and *Q*–*Q* plots of within‐group change scores and ANCOVA residuals; no material departures were identified for the primary models. Baseline characteristics were compared using one‐way ANOVA or chi‐square tests. Within‐group paired *t*‐tests were descriptive. The primary between‐group analysis used ANCOVA with the Week 12 value as the dependent variable, treatment group as the fixed factor and the corresponding baseline value as the covariate. Prespecified pairwise contrasts compared TCE versus control, KEE versus control and TCE versus KEE. Secondary outcomes were exploratory. Descriptive responder analyses counted participants with any favourable within‐person change (decrease in FPG, 2‐h OGTT glucose, or HbA1c; increase in 25(OH)D_3_) and were not treated as prespecified efficacy endpoints. Partial correlations between changes in 25(OH)D_3_ and changes in metabolic outcomes were adjusted for age, sex, baseline BMI and treatment group; group‐specific analyses adjusted for age, sex and baseline BMI. Two‐sided *p* < 0.05 was considered statistically significant.

## 3 Results

### 3.1 Participant Flow and Baseline Characteristics

**FIGURE 1:**
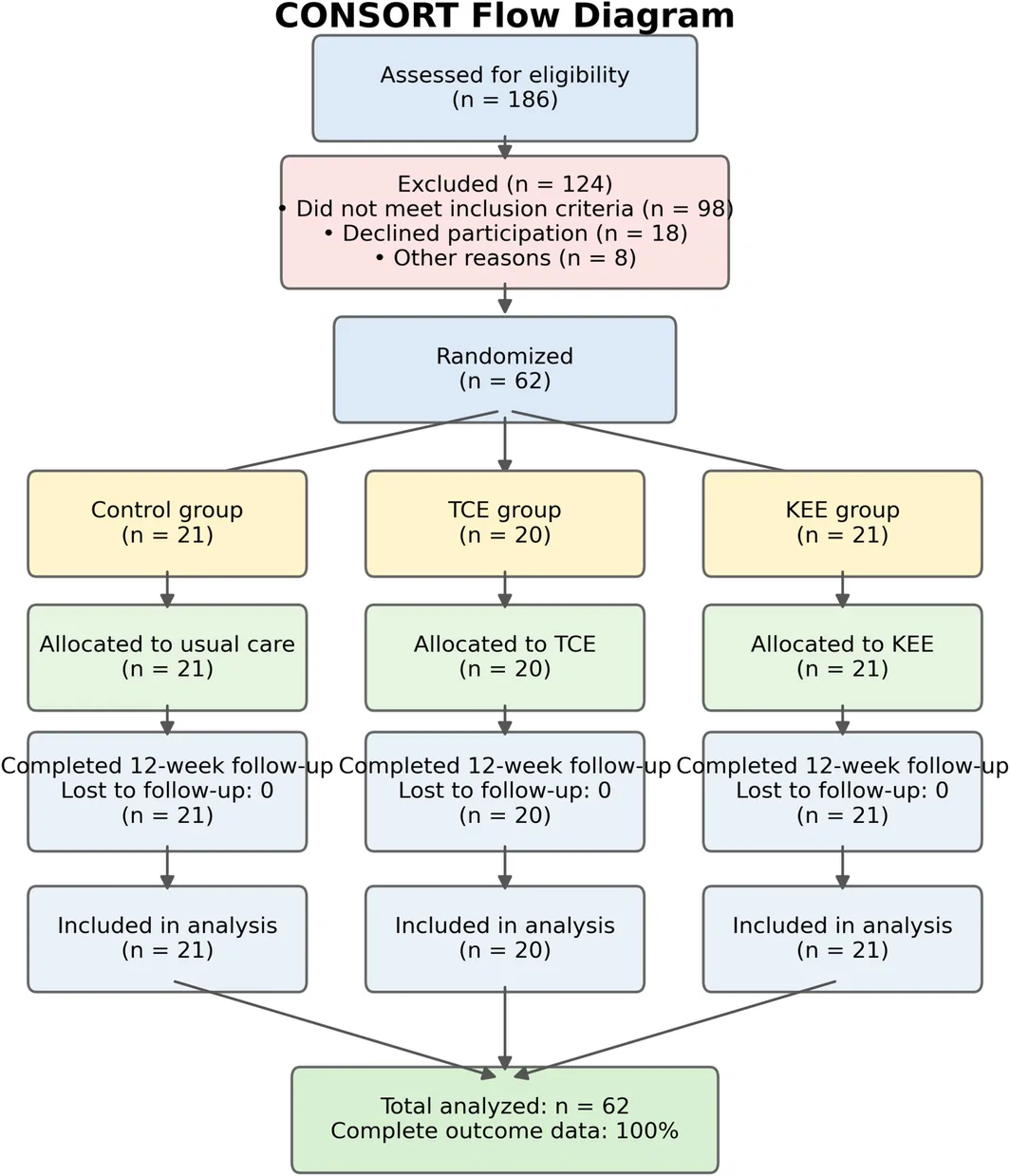
CONSORT flow diagram showing participant screening, randomisation, allocation, 12‐week follow‐up and analysis. KEE, Kuanle Equipment Exercise; TCE, Traditional Chinese Exercise.

Between July 2024 and December 2024, 186 individuals were assessed for eligibility, of whom 124 were excluded (98 did not meet inclusion criteria, 18 declined participation, 8 for other reasons). Sixty‐two participants were randomised: 21 to control, 20 to TCE and 21 to KEE groups. All 62 participants completed the 12‐week intervention with 100% data completeness and no loss to follow‐up (Figure 1). This high retention rate was supported by monthly telephone follow‐ups, weekly attendance reminders for exercise groups, flexible scheduling and community‐based recruitment from a stable local population.

**FIGURE 2:**
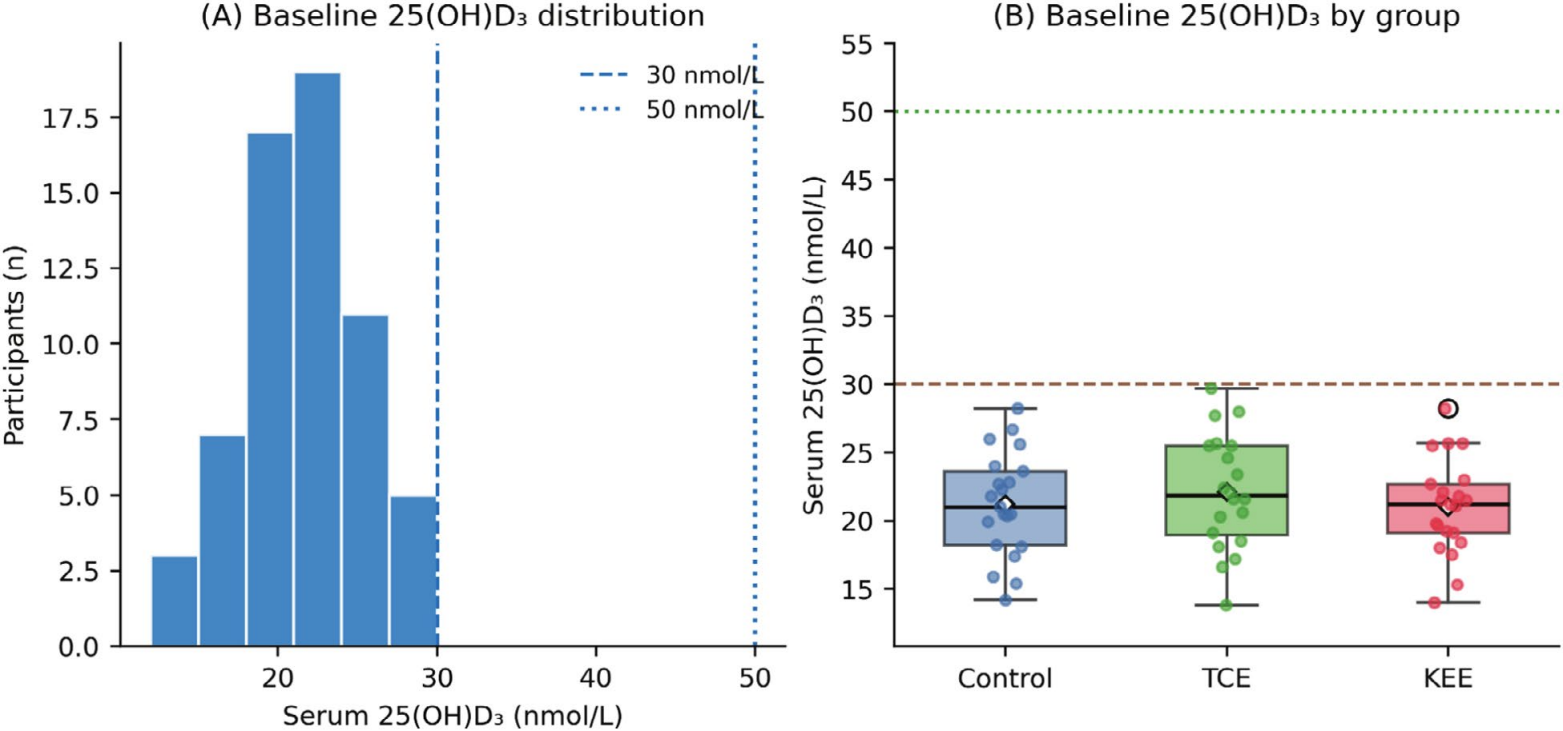
Baseline serum 25(OH)D_3_ concentrations. (A) Distribution among all participants (*n* = 62), with reference lines at 30 and 50 nmol/L. (B) Group‐specific box‐and‐point plots. Boxes show the interquartile range, centre lines the median, diamonds the mean and whiskers 1.5 × IQR.

Baseline characteristics were well‐balanced across groups (Table 1). The cohort comprised predominantly female participants (88.7%, *n* = 55) with a mean age of 55.2 ± 8.4 years. Age distribution included 83.9% (*n* = 52) aged < 60 years, 12.9% (*n* = 8) aged 60–69 years and 3.2% (*n* = 2) aged 70–79 years. Mean BMI was 25.8 ± 3.2 kg/m^2^, consistent with overweight status. According to Chinese BMI cut‐offs, 37.1% (*n* = 23) were overweight (24–27.9 kg/m^2^) and 35.5% (*n* = 22) were obese (≥ 28 kg/m^2^). Prediabetes criteria fulfilment included isolated impaired fasting glucose (41.9%, *n* = 26), isolated impaired glucose tolerance (22.6%, *n* = 14), elevated HbA1c (12.9%, *n* = 8) and combined criteria (22.6%, *n* = 14). Notably, all participants exhibited vitamin D deficiency, with a mean 25(OH)D_3_ of 21.53 ± 3.90 nmol/L—well below the sufficiency threshold of 50 nmol/L. The distribution of baseline 25(OH)D_3_ concentrations across all participants and by group is shown in Figure 2.

**TABLE 1:** Baseline characteristics of study participants.

Characteristic	Control (*n* = 21)	TCE (*n* = 20)	KEE (*n* = 21)	Total (*n* = 62)	*p*
Age (years)	55.1 ± 8.6	54.8 ± 8.2	55.7 ± 8.5	55.2 ± 8.4	0.942
Female, *n* (%)	19 (90.5)	17 (85.0)	19 (90.5)	55 (88.7)	0.798
BMI (kg/m^2^)	25.9 ± 3.1	25.6 ± 3.0	25.9 ± 3.5	25.8 ± 3.2	0.947
*BMI category, n (%)*					0.891
< 24 kg/m^2^	6 (28.6)	6 (30.0)	5 (23.8)	17 (27.4)	
24–27.9 kg/m^2^	8 (38.1)	7 (35.0)	8 (38.1)	23 (37.1)	
≥ 28 kg/m^2^	7 (33.3)	7 (35.0)	8 (38.1)	22 (35.5)	
FPG (mmol/L)	6.5 ± 0.4	6.4 ± 0.5	6.6 ± 0.5	6.5 ± 0.5	0.412
2 h OGTT (mmol/L)	9.8 ± 1.2	9.6 ± 1.3	9.9 ± 1.4	9.8 ± 1.3	0.723
HbA1c (%)	6.1 ± 0.3	6.0 ± 0.4	6.2 ± 0.4	6.1 ± 0.4	0.289
25(OH)D_3_ (nmol/L)	21.2 ± 3.8	22.1 ± 4.2	21.0 ± 3.5	21.5 ± 3.9	0.651
TG (mmol/L)	2.1 ± 0.4	2.0 ± 0.5	2.1 ± 0.4	2.1 ± 0.4	0.783
TC (mmol/L)	5.4 ± 0.6	5.3 ± 0.7	5.4 ± 0.6	5.4 ± 0.6	0.892
*Prediabetes criteria, n (%)*					0.754
Isolated IFG	9 (42.9)	8 (40.0)	9 (42.9)	26 (41.9)	
Isolated IGT	4 (19.0)	5 (25.0)	5 (23.8)	14 (22.6)	
Elevated HbA1c	3 (14.3)	2 (10.0)	3 (14.3)	8 (12.9)	
Combined criteria	5 (23.8)	5 (25.0)	4 (19.0)	14 (22.6)	

*Note:* Values are presented as mean ± SD or *n* (%).

Abbreviations: 25(OH)D_3_, 25‐hydroxyvitamin D_3_; BMI, body mass index; FPG, fasting plasma glucose; HbA1c, glycated haemoglobin; IFG, impaired fasting glucose; IGT, impaired glucose tolerance; OGTT, oral glucose tolerance test; TC, total cholesterol; TG, triglycerides.

### 3.2 Secondary Outcomes (Lipid Profiles and Body Composition)

Compared with control after adjustment for baseline values, both TCE and KEE were associated with lower Week 12 triglycerides (*p* = 0.002 and *p* < 0.001, respectively), total cholesterol (*p* = 0.022 and *p* = 0.007) and LDL‐C (both *p* < 0.001) (Table 2). HDL‐C did not differ significantly from control after baseline adjustment (TCE *p* = 0.553; KEE *p* = 0.056). Direct TCE–KEE comparisons were not statistically significant for any lipid outcome (*p* = 0.115–0.671).

**TABLE 2:** Secondary outcomes at baseline and 12 weeks.

Outcome	Group	Baseline	12 weeks	Change (%)	*p* *	*p* ^†^	*p* ^‡^
TG (mmol/L)	Control	2.10 ± 0.40	2.05 ± 0.40	−2.4	0.488	—	—
	TCE	2.00 ± 0.50	1.70 ± 0.40	−15.0	0.002	0.002	0.314
	KEE	2.10 ± 0.40	1.68 ± 0.40	−20.0	< 0.001	< 0.001	
TC (mmol/L)	Control	5.40 ± 0.60	5.30 ± 0.60	−1.9	0.269	—	—
	TCE	5.30 ± 0.70	4.90 ± 0.60	−7.5	0.005	0.022	0.671
	KEE	5.40 ± 0.60	4.90 ± 0.60	−9.3	< 0.001	0.007	
LDL‐C (mmol/L)	Control	3.20 ± 0.35	3.16 ± 0.35	−1.3	0.511	—	—
	TCE	3.15 ± 0.35	2.83 ± 0.35	−10.2	< 0.001	< 0.001	0.115
	KEE	3.20 ± 0.35	2.75 ± 0.35	−14.1	< 0.001	< 0.001	
HDL‐C (mmol/L)	Control	1.25 ± 0.15	1.26 ± 0.15	+0.8	0.654	—	—
	TCE	1.20 ± 0.15	1.25 ± 0.15	+4.2	0.112	0.553	0.196
	KEE	1.25 ± 0.15	1.33 ± 0.15	+6.4	0.019	0.056	
BMI (kg/m^2^)	Control	25.90 ± 3.10	25.80 ± 3.00	−0.4	0.572	—	—
	TCE	25.60 ± 3.00	24.70 ± 2.90	−3.5	0.004	0.003	0.305
	KEE	25.90 ± 3.50	24.70 ± 3.30	−4.6	< 0.001	< 0.001	
Body fat (%)	Control	32.50 ± 4.20	32.30 ± 4.10	−0.6	0.557	—	—
	TCE	32.10 ± 4.00	30.40 ± 3.80	−5.3	0.003	0.002	0.255
	KEE	32.60 ± 4.50	30.30 ± 4.20	−7.1	< 0.001	< 0.001	
Skeletal muscle mass (kg)	Control	22.50 ± 1.80	22.46 ± 1.80	−0.2	0.730	—	—
	TCE	22.20 ± 1.80	22.70 ± 1.80	+2.3	0.012	0.037	0.347
	KEE	22.50 ± 1.80	23.20 ± 1.80	+3.1	0.002	0.003	

* Within‐group paired *t*‐test.

^†^ ANCOVA contrast versus control, with Week 12 value as the dependent variable and the corresponding baseline value as covariate.

^‡^ Direct TCE–KEE ANCOVA contrast. Secondary‐outcome analyses were exploratory.

Both exercise groups also showed favourable body‐composition changes. After baseline adjustment, BMI was lower than control in TCE (*p* = 0.003) and KEE (*p* < 0.001), as was body fat percentage (*p* = 0.002 and *p* < 0.001). Skeletal muscle mass was higher than control in TCE (*p* = 0.037) and KEE (*p* = 0.003). Direct TCE–KEE comparisons were not statistically significant for BMI, body fat, or skeletal muscle mass (all *p* ≥ 0.255).

### 3.3 Primary Outcomes (Glucose Metabolism and Vitamin D)

#### 3.3.1 Glucose Metabolism Parameters

**FIGURE 3:**
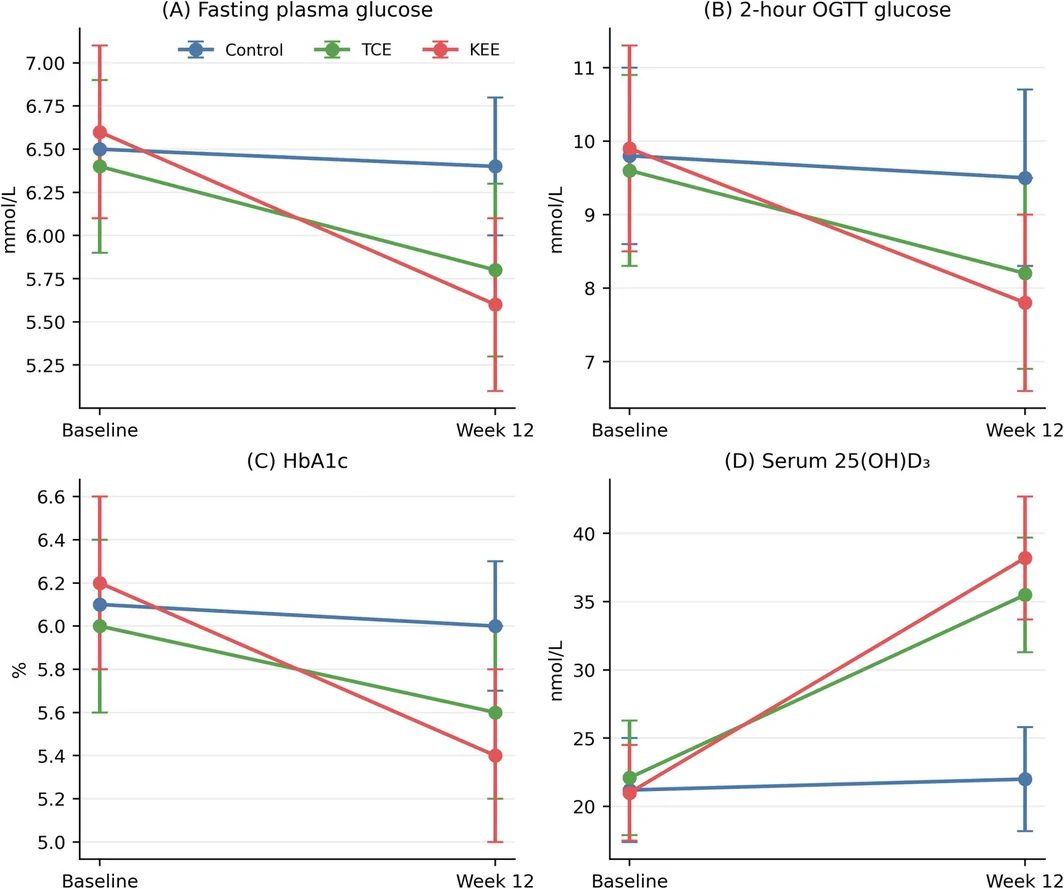
Primary outcomes at baseline and Week 12. Points show group means and error bars show SD for (A) FPG, (B) 2‐h OGTT glucose, (C) HbA1c and (D) serum 25(OH)D_3_. Formal between‐group inference was based on ANCOVA adjusted for the corresponding baseline outcome (Table 3).

At 12 weeks, both exercise interventions showed significant baseline‐adjusted differences from control in FPG, 2‐h OGTT glucose, HbA1c and 25(OH)D_3_ (Table 3; Figure 3).

**TABLE 3:** Primary outcomes at baseline and 12 weeks.

Outcome	Group	Baseline	12 weeks	Change (%)	*p* *	*p* ^†^	*p* ^‡^
FPG (mmol/L)	Control	6.5 ± 0.4	6.4 ± 0.4	−1.5	0.312	—	—
	TCE	6.4 ± 0.5	5.8 ± 0.5	−9.4	< 0.001	< 0.001	0.078
	KEE	6.6 ± 0.5	5.6 ± 0.5	−15.2	< 0.001	< 0.001	
2 h OGTT (mmol/L)	Control	9.8 ± 1.2	9.5 ± 1.2	−3.1	0.285	—	—
	TCE	9.6 ± 1.3	8.2 ± 1.3	−14.6	< 0.001	< 0.001	0.071
	KEE	9.9 ± 1.4	7.8 ± 1.2	−21.2	< 0.001	< 0.001	
HbA1c (%)	Control	6.1 ± 0.3	6.0 ± 0.3	−1.6	0.246	—	—
	TCE	6.0 ± 0.4	5.6 ± 0.4	−6.7	< 0.001	0.001	0.089
	KEE	6.2 ± 0.4	5.4 ± 0.4	−12.9	< 0.001	< 0.001	
25(OH)D_3_ (nmol/L)	Control	21.2 ± 3.8	22.0 ± 3.8	+3.8	0.412	—	—
	TCE	22.1 ± 4.2	35.5 ± 4.2	+60.6	< 0.001	< 0.001	0.073
	KEE	21.0 ± 3.5	38.2 ± 4.5	+81.9	< 0.001	< 0.001	

* Within‐group paired *t*‐test.

^†^ ANCOVA contrast versus control, with Week 12 value as the dependent variable and the corresponding baseline value as covariate.

^‡^ Direct TCE–KEE ANCOVA contrast.

Mean FPG changed from 6.4 ± 0.5 to 5.8 ± 0.5 mmol/L in TCE and from 6.6 ± 0.5 to 5.6 ± 0.5 mmol/L in KEE, compared with 6.5 ± 0.4 to 6.4 ± 0.4 mmol/L in control. Baseline‐adjusted differences versus control were significant for TCE and KEE (both *p* < 0.001), whereas the direct TCE–KEE comparison was not significant (*p* = 0.078). However, the observed numerical differences between the two exercise groups should be interpreted with caution given the lack of statistical significance and the reduced statistical power for the head‐to‐head comparison.

Mean 2‐h OGTT glucose changed from 9.6 ± 1.3 to 8.2 ± 1.3 mmol/L in TCE and from 9.9 ± 1.4 to 7.8 ± 1.2 mmol/L in KEE, compared with 9.8 ± 1.2 to 9.5 ± 1.2 mmol/L in control. Baseline‐adjusted differences versus control were significant for both exercise groups (both *p* < 0.001); the direct TCE–KEE comparison was not significant (*p* = 0.071).

Mean HbA1c changed from 6.0% ± 0.4% to 5.6% ± 0.4% in TCE and from 6.2% ± 0.4% to 5.4% ± 0.4% in KEE, compared with 6.1% ± 0.3% to 6.0% ± 0.3% in control. Baseline‐adjusted differences versus control were significant for TCE (*p* = 0.001) and KEE (*p* < 0.001); the direct TCE–KEE comparison was not significant (*p* = 0.089).

#### 3.3.2 Vitamin D Levels

Serum 25(OH)D_3_ increased from 22.1 ± 4.2 to 35.5 ± 4.2 nmol/L in TCE and from 21.0 ± 3.5 to 38.2 ± 4.5 nmol/L in KEE, compared with 21.2 ± 3.8 to 22.0 ± 3.8 nmol/L in control (Figure 3D). Baseline‐adjusted comparisons favoured both exercise groups over control (both *p* < 0.001). The direct TCE–KEE comparison did not reach statistical significance (*p* = 0.073). Mean Week 12 concentrations remained below 50 nmol/L in both exercise groups.

#### 3.3.3 Clinical Response Rates

In a descriptive, non‐prespecified responder analysis, any favourable change from baseline was observed for FPG in 12/21 (57.1%) control, 16/20 (80.0%) TCE and 19/21 (90.5%) KEE participants; for 2‐h OGTT glucose in 12/21 (57.1%), 19/20 (95.0%) and 20/21 (95.2%), respectively; for HbA1c in 12/21 (57.1%), 15/20 (75.0%) and 18/21 (85.7%); and for 25(OH)D_3_ in 13/21 (61.9%), 19/20 (95.0%) and 21/21 (100.0%).

### 3.4 Correlation Analysis

**FIGURE 4:**
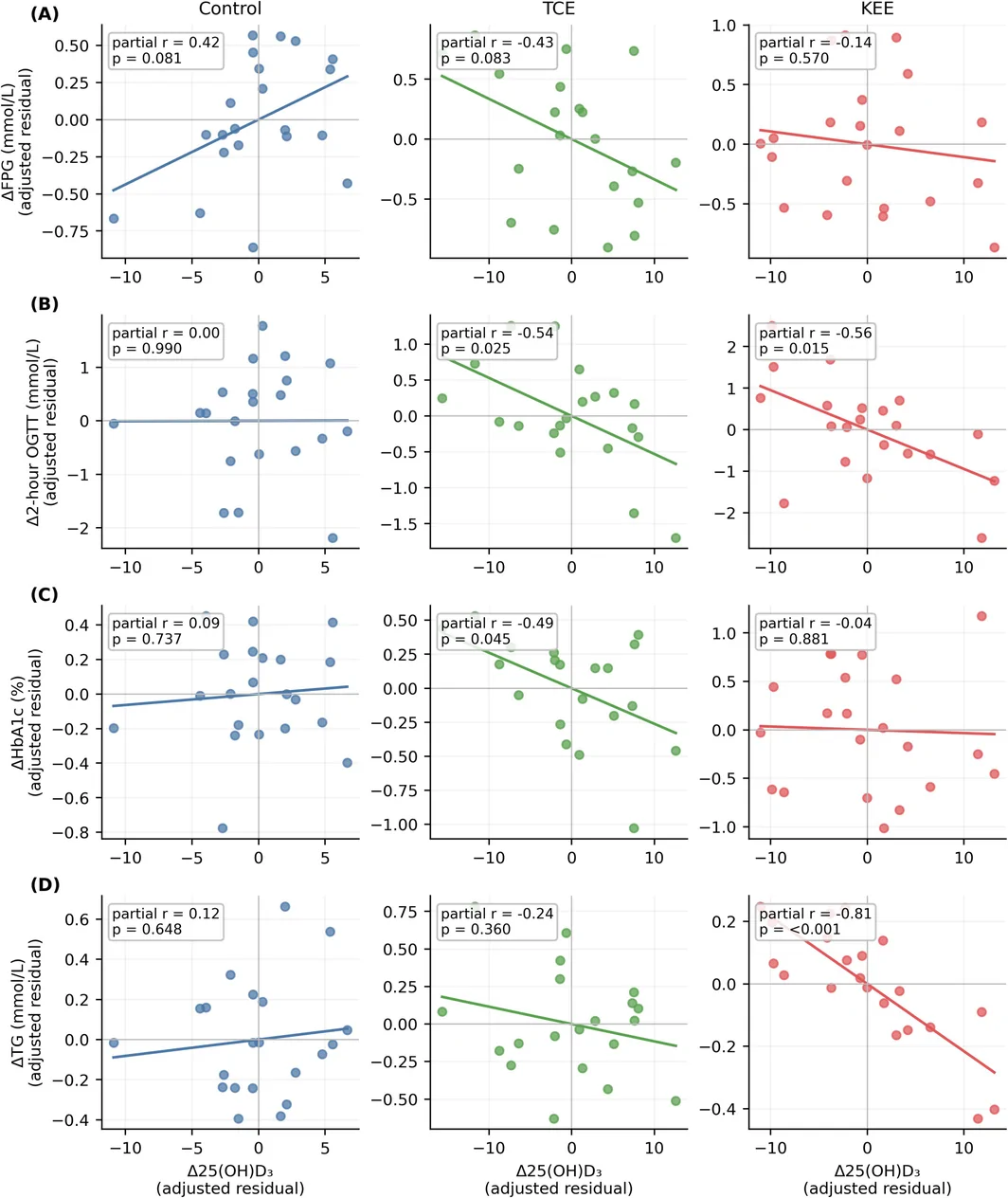
Exploratory group‐specific partial correlations between changes in serum 25(OH)D_3_ and changes in metabolic outcomes. Panels show covariate‐adjusted residuals within each group for (A) FPG, (B) 2‐h OGTT glucose, (C) HbA1c and (D) triglycerides. Group‐specific analyses adjusted for age, sex and baseline BMI; fitted lines are shown for visualisation.

Exploratory partial correlations examined within‐person change scores. Across the full cohort, after adjustment for age, sex, baseline BMI and treatment group, change in 25(OH)D_3_ was inversely associated with change in 2‐h OGTT glucose (partial *r* = −0.358, *p* = 0.006), but not with change in FPG (*r* = −0.185, *p* = 0.168), HbA1c (*r* = −0.122, *p* = 0.365), or triglycerides (*r* = −0.210, *p* = 0.117). Group‐specific exploratory analyses were heterogeneous and did not show a consistent association across both exercise groups (Figure 4).

### 3.5 Safety and Adverse Events

Both exercise interventions were well‐tolerated with no serious adverse events reported. Minor musculoskeletal complaints occurred in 3 TCE participants (transient knee discomfort, *n* = 2; mild lower back soreness, *n* = 1) and 2 KEE participants (shoulder strain, *n* = 1; calf muscle soreness, *n* = 1), all resolving within 1 week with modified exercise progression. No hypoglycaemic events occurred during the study period.

## 4 Discussion

### 4.1 Principal Findings

This randomised controlled trial found that both TCE and KEE improved glycaemic outcomes and increased serum 25(OH)D_3_ concentrations compared with usual care over 12 weeks. KEE produced numerically larger mean changes, but direct TCE–KEE comparisons were not statistically significant. Exploratory change‐score analyses identified an inverse association between changes in 25(OH)D_3_ and 2‐h OGTT glucose, but not with the other metabolic outcomes examined; therefore, the data do not establish vitamin D as a mediator of the glycaemic effects.

### 4.2 Comparison With Previous Studies

Our findings align with prior evidence supporting exercise for prediabetes management. The Diabetes Prevention Program demonstrated that lifestyle intervention reduced diabetes incidence by 58% over 3 years [3]. More recently, a meta‐analysis of 47 randomised trials in patients with type 2 diabetes mellitus confirmed that structured exercise reduces HbA1c by approximately 0.6% [17]. While this evidence pertains to T2DM, the magnitude of effect is consistent with our findings in prediabetic participants, suggesting consistent metabolic responsiveness to exercise across the dysglycemia spectrum. Our observed HbA1c reductions of 0.4%–0.8% over 12 weeks are consistent with these estimates. However, whether longer intervention periods would yield further improvements or potentially widen the between‐group differences remains unknown and requires future investigation.

KEE showed numerically larger mean changes than TCE, which is biologically plausible because the intervention combined aerobic and resistance components. Prior evidence suggests that combined aerobic and resistance training may improve glycaemic control [18]. Nevertheless, the direct TCE–KEE contrasts in the present trial were not statistically significant for primary outcomes (*p* = 0.071–0.089 for glycaemic outcomes and *p* = 0.073 for 25(OH)D_3_). These findings should therefore be interpreted as descriptive and hypothesis‐generating rather than evidence of KEE superiority.

### 4.3 Exercise‐Induced Vitamin D Improvements

The increase in serum 25(OH)D_3_ observed in both exercise groups is noteworthy but requires cautious interpretation. Crucially, since all exercise sessions were conducted indoors at the Health Management Center (see Section 2.4.4), the observed increases in serum 25(OH)D_3_ in the exercise groups cannot be attributed to differential sunlight exposure. This supports the hypothesis that exercise‐induced metabolic adaptations—such as reduced adipose tissue sequestration of vitamin D or altered hepatic hydroxylation—may contribute, though this remains speculative without direct mechanistic assays. Nevertheless, the trial did not quantify dietary vitamin D intake, clothing coverage, sunscreen use, or seasonal variation and the intervention spanned July through December. Thus, while we can confidently exclude differential sunlight exposure as a driver of the observed increase, the relative contributions of exercise per se versus other unmeasured behavioural or metabolic factors remain unclear; the proposed mechanisms [14, 19] await confirmation in dedicated mechanistic studies.

The exploratory correlation analysis provided limited support for a direct mechanistic link. After adjustment for treatment group and participant characteristics, change in 25(OH)D_3_ was associated with change in 2‐h OGTT glucose but not with changes in FPG, HbA1c, or triglycerides. Because these analyses were secondary, based on a small sample and potentially affected by residual confounding, they should not be interpreted as evidence of mediation or causality.

### 4.4 Clinical Implications

These findings support structured exercise as an important component of prediabetes management in middle‐aged and elderly adults. TCE offers a culturally familiar, low‐impact option, whereas KEE provides a standardised combination of aerobic and resistance exercise. The concomitant increase in 25(OH)D_3_ is potentially relevant but should not replace established assessment and treatment of vitamin D deficiency.

However, it is noteworthy that despite substantial improvements, mean vitamin D levels remained below sufficiency thresholds. This suggests that exercise alone may be insufficient for achieving optimal vitamin D levels, and supplementation may still be warranted, particularly during winter months or for individuals with limited sun exposure.

### 4.5 Strengths and Limitations

Strengths of this study include the randomised controlled design, high completion rate (100%) and comprehensive metabolic assessment. The comparison of two distinct exercise modalities provides practical guidance for exercise prescription.

Limitations should be acknowledged. First, the final sample (*n* = 62) was smaller than the planned *n* = 90, limiting precision for smaller effects, direct TCE–KEE comparisons and subgroup analyses. Second, the 12‐week intervention does not establish long‐term durability. Third, dietary vitamin D intake and sunlight exposure—including time outdoors, clothing coverage, season and sunscreen use—were not systematically measured; these omissions are particularly important because recruitment and follow‐up occurred from July to December. Fourth, 88.7% of participants were female, limiting generalisability to men. Fifth, the trial was conducted at a single centre and was not prospectively registered in a public trial registry. Sixth, the duration of prediabetes was not systematically recorded. Finally, responder and correlation analyses were exploratory and not prespecified; the correlation findings may be sensitive to treatment‐group effects and residual confounding.

### 4.6 Future Directions

Future research should address several questions: (1) longer‐term studies assessing sustainability and durability of metabolic improvements; (2) mechanistic investigations into exercise‐induced vitamin D metabolism changes; (3) head‐to‐head powered comparisons of TCE versus KEE with formal statistical testing; (4) studies comparing exercise alone versus exercise plus vitamin D supplementation; and (5) cost‐effectiveness analyses to inform healthcare policy decisions regarding exercise prescription for prediabetes.

## 5 Conclusion

In this 12‐week randomised trial, both TCE and KEE improved glycaemic outcomes and increased serum 25(OH)D_3_ concentrations compared with usual care in middle‐aged and elderly adults with prediabetes. KEE produced numerically larger mean changes, but superiority over TCE was not established. The vitamin D finding and its relationship with glucose metabolism should be confirmed in larger, prospectively registered studies that measure sunlight exposure, dietary vitamin D and longer‐term outcomes.

## Author Contributions

**Xu Lingzhi:** conceptualization, methodology, writing – original draft, writing – review and editing, data curation. **Yuan Yimin:** funding acquisition, project administration, resources, supervision, writing – review and editing. **Liu Wu:** investigation, data curation. **Ding Jiejin:** conceptualization. **Zhou Chunhua:** data curation, writing – review and editing, investigation.

## Funding

This work was supported by the Wuxi Hospital of Traditional Chinese Medicine Research Fund.

## Ethics Statement

The study protocol was approved by the Institutional Review Board of Wuxi Hospital of Traditional Chinese Medicine (IRB approval number: 20240326). All participants provided written informed consent. The trial was filed with the National Medical Research Registration and Filing Information System (MR‐32‐24‐047398) before enrollment; retrospective registration with ChiCTR is pending review.

## Consent

All participants provided informed consent for the publication of anonymised data. No individually identifiable information is presented in this manuscript.

## Conflicts of Interest

The authors declare no conflicts of interest.

## Supporting information


**File S1:** National medical research registration and filing information system filing record (MR‐32‐24‐047398). Available at: https://www.medicalresearch.org.cn/.

## Data Availability

The datasets generated and analysed during the current study are available from the corresponding author on reasonable request.
