# Supplementary material for: Comparison of Traditional Chinese Exercise and Equipment‐Based Exercise on Glycaemic Control and Vitamin D Levels in Middle‐Aged and Elderly Patients With Prediabetes: A Randomised Controlled Trial
Source: Endocrinol Diabetes Metab. 2026 Sep 8;9(5):e70326. doi: 10.1002/edm2.70326 (PMC13552993; doi:10.1002/edm2.70326)
Supplement: Supplementary file 1 [file EDM2-9-e70326-s001.pdf]

Comparison of Traditional Chinese Exercise and Equipment-based Exercise on Glycemic Control and Vitamin D Levels in Middle-aged and Elderly Patients with Prediabetes: A Randomized Controlled Trial

Filing Number: MR-32-24-047398

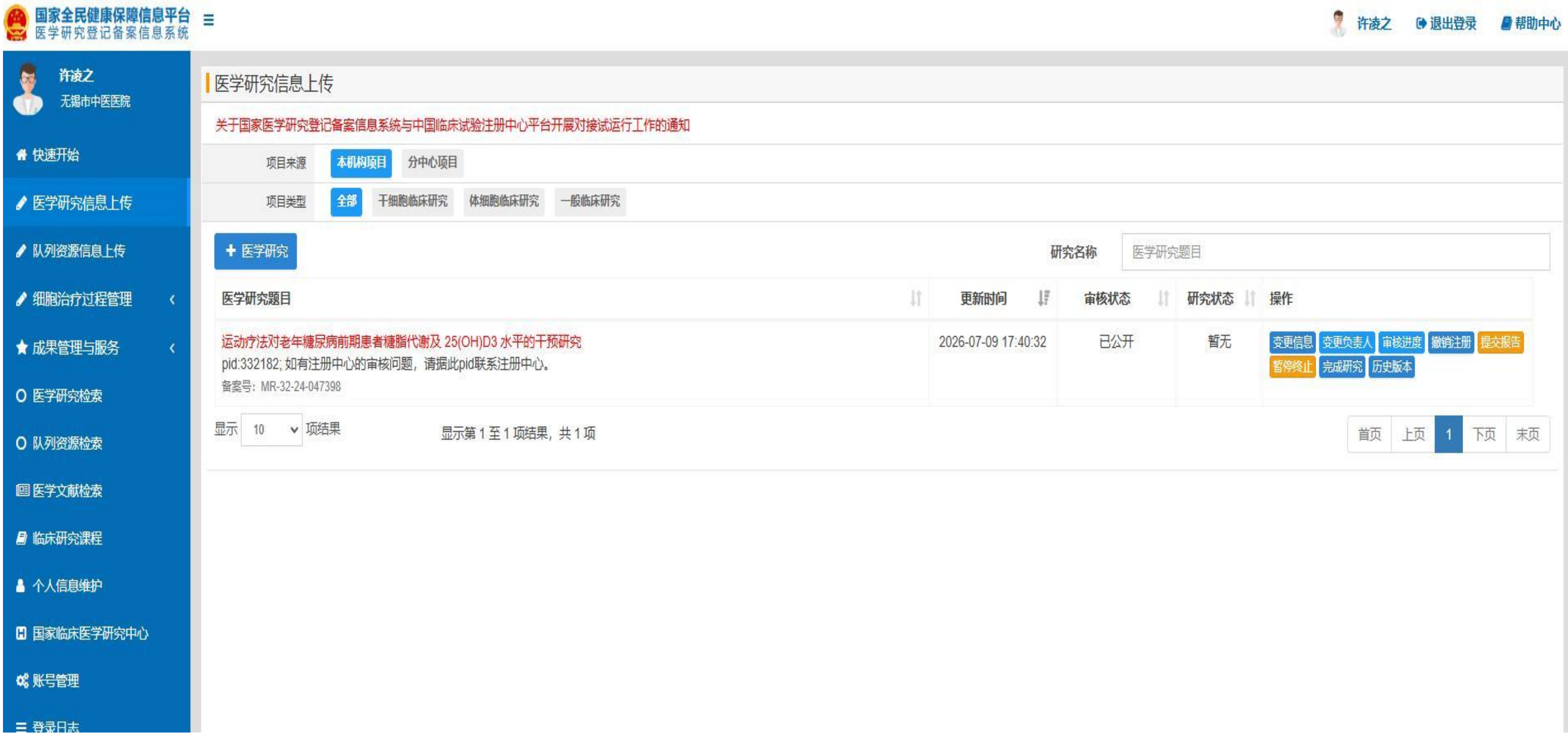
国家全民健康保障信息平台
医学研究登记备案信息系统
许凌之 退出登录 帮助中心

许凌之
无锡市中医医院
快速开始
医学研究信息上传
队列资源信息上传
细胞治疗过程管理
成果管理与服务
医学研究检索
队列资源检索
医学文献检索
临床研究课程
个人信息维护
国家临床医学研究中心
账号管理
登录日志

医学研究信息上传

关于国家医学研究登记备案信息系统与中国临床试验注册中心平台开展对接试运行工作的通知

项目来源 本机构项目 分中心项目
项目类型 全部 干细胞临床研究 体细胞临床研究 一般临床研究

+ 医学研究 研究名称 医学研究题目

医学研究题目	更新时间	审核状态	研究状态	操作
运动疗法对老年糖尿病前期患者糖脂代谢及 25(OH)D3 水平的干预研究<br>pid:332182; 如有注册中心的审核问题，请据此pid联系注册中心。<br>备案号：MR-32-24-047398	2026-07-09 17:40:32	已公开	暂无	变更信息 变更负责人 审核进度 撤销注册 提交报告 暂停终止 完成研究 历史版本

显示 10 项结果 显示第 1 至 1 项结果，共 1 项
首页 上页 1 下页 末页

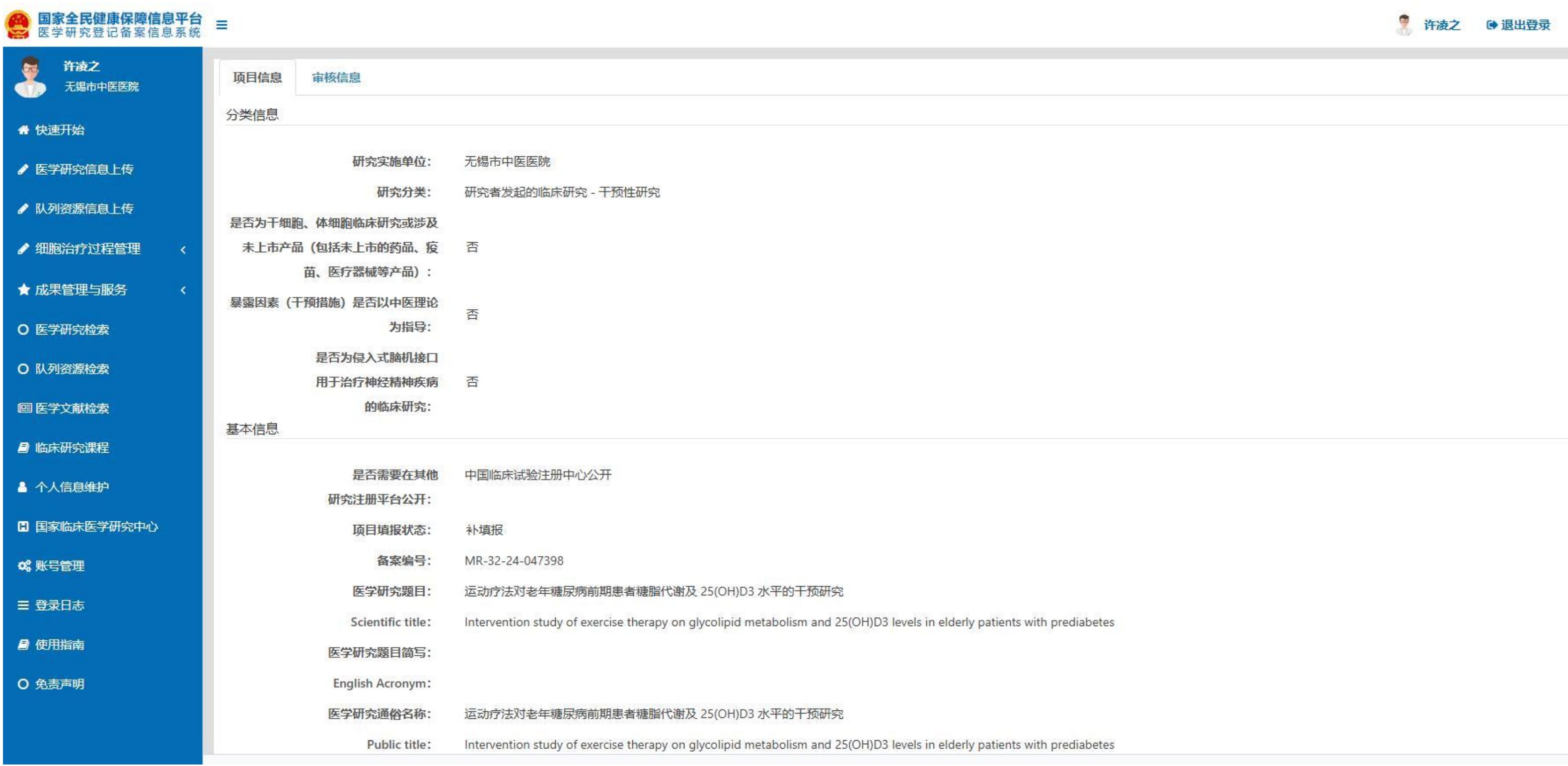
国家全民健康保障信息平台
医学研究登记备案信息系统
许凌之 退出登录

许凌之
无锡市中医医院
快速开始
医学研究信息上传
队列资源信息上传
细胞治疗过程管理
成果管理与服务
医学研究检索
队列资源检索
医学文献检索
临床研究课程
个人信息维护
国家临床医学研究中心
账号管理
登录日志
使用指南
免责声明

项目信息 审核信息

分类信息

研究实施单位： 无锡市中医医院
研究分类： 研究者发起的临床研究 - 干预性研究
是否为干细胞、体细胞临床研究或涉及未上市产品（包括未上市的药品、疫苗、医疗器械等产品）： 否
暴露因素（干预措施）是否以中医理论为指导： 否
是否为侵入式脑机接口用于治疗神经精神疾病的临床研究： 否

基本信息

是否需要在其他研究注册平台公开： 中国临床试验注册中心公开
项目填报状态： 补填报
备案编号： MR-32-24-047398
医学研究题目： 运动疗法对老年糖尿病前期患者糖脂代谢及 25(OH)D3 水平的干预研究
Scientific title： Intervention study of exercise therapy on glycolipid metabolism and 25(OH)D3 levels in elderly patients with prediabetes
医学研究题目简写：
English Acronym：
医学研究通俗名称： 运动疗法对老年糖尿病前期患者糖脂代谢及 25(OH)D3 水平的干预研究
Public title： Intervention study of exercise therapy on glycolipid metabolism and 25(OH)D3 levels in elderly patients with prediabetes
